# Retraction: ZNF655 promotes the progression of glioma through transcriptional regulation of AURKA

**DOI:** 10.3389/fonc.2023.1294900

**Published:** 2023-10-30

**Authors:** Frontiers Editorial Office

Following publication, concerns were raised regarding the validity of the data in the article. Following investigation, which was conducted in accordance with Frontiers’ policies, the Chief Editor concluded that the article’s conclusions and assertions were not sufficiently supported by the findings from the material provided; therefore, the article has been retracted. This retraction was approved by the Chief Editors of Frontiers in Oncology.

The authors did not agree with the retraction.

